# A platform independent RNA-Seq protocol for the detection of transcriptome complexity

**DOI:** 10.1186/1471-2164-14-855

**Published:** 2013-12-05

**Authors:** Claudia Calabrese, Marina Mangiulli, Caterina Manzari, Anna Maria Paluscio, Mariano Francesco Caratozzolo, Flaviana Marzano, Ivana Kurelac, Anna Maria D’Erchia, Domenica D’Elia, Flavio Licciulli, Sabino Liuni, Ernesto Picardi, Marcella Attimonelli, Giuseppe Gasparre, Anna Maria Porcelli, Graziano Pesole, Elisabetta Sbisà, Apollonia Tullo

**Affiliations:** Dip. Scienze Mediche e Chirurgiche (DIMEC), U.O. Genetica Medica, Pol. Universitario S. Orsola-Malpighi, Università di Bologna, via Massarenti 9, 40138 Bologna, Italy; Dip. Bioscienze, Biotecnologie e Biofarmaceutica, Università di Bari, via E. Orabona 4, 70126 Bari, Italy; Istituto di Tecnologie Biomediche (ITB), Consiglio Nazionale delle Ricerche (CNR), Bari, Italy; Dip. Farmacia e Biotecnologie (FABIT), Università di Bologna, Bologna, Italy; Istituto di Biomembrane e Bioenergetica (IBBE), Consiglio Nazionale delle Ricerche (CNR), Bari, Italy

**Keywords:** RNA-seq, cDNA library preparation, Platform-independent RNA-seq

## Abstract

**Background:**

Recent studies have demonstrated an unexpected complexity of transcription in eukaryotes. The majority of the genome is transcribed and only a little fraction of these transcripts is annotated as protein coding genes and their splice variants. Indeed, most transcripts are the result of antisense, overlapping and non-coding RNA expression. In this frame, one of the key aims of high throughput transcriptome sequencing is the detection of all RNA species present in the cell and the first crucial step for RNA-seq users is represented by the choice of the strategy for cDNA library construction. The protocols developed so far provide the utilization of the entire library for a single sequencing run with a specific platform.

**Results:**

We set up a unique protocol to generate and amplify a strand-specific cDNA library representative of all RNA species that may be implemented with all major platforms currently available on the market (Roche 454, Illumina, ABI/SOLiD). Our method is reproducible, fast, easy-to-perform and even allows to start from low input total RNA. Furthermore, we provide a suitable bioinformatics tool for the analysis of the sequences produced following this protocol.

**Conclusion:**

We tested the efficiency of our strategy, showing that our method is platform-independent, thus allowing the simultaneous analysis of the same sample with different NGS technologies, and providing an accurate quantitative and qualitative portrait of complex whole transcriptomes.

**Electronic supplementary material:**

The online version of this article (doi:10.1186/1471-2164-14-855) contains supplementary material, which is available to authorized users.

## Background

Next Generation Sequencing (NGS) technology, initially used almost exclusively to sequence and/or re-sequence whole genomes and to determine single nucleotide polymorphisms (SNP) [1], is nowadays increasingly exploited in an effective manner for many other applications, including identification of biological markers and pathogenic agents in biodiversity studies and metagenomics, in biomedical, agro-food, environmental and industrial sectors [2–4].

Interestingly, quite recently the ENCODE Project (Encyclopedia of DNA Elements) has revealed that the vast majority of human genome is pervasively transcribed and almost the totality of the DNA is associated to some biochemical RNA-associated function [5]. Indeed, the transcriptome captures a level of complexity that the simple genome sequence does not [6]. In this frame, the RNA-seq technology is an effective method to map functional elements across the human genome and a suitable tool for the analysis of genome-wide differential RNA expression, underlining the wide range of physical, epigenetics, biochemical, and developmental differences observed among various cells and tissues, that may play a role in determining the balance between health and disease [7, 8].

Among next generation sequencing platforms, the latest series of Roche 454 GS Sequencer, the GS FLX Titanium FLX+, allows to obtain over a million reads in each run, with a length up to 700 bases [9]. Such long reads provide connectivity information among splicing sites, in addition to enabling accurate mapping and relative quantification of mRNAs, hence they are particularly suitable for the characterization of full-length splicing variants that may be differently expressed in physiopathological conditions [7, 10]. On the other hand, the higher throughput, although with shorter reads, of the Illumina and ABI/SOLiD platforms makes them particularly suitable to better quantify the levels of transcripts and for small RNAs sequencing [11–13].

Irrespectively of the NGS platform used, the first step required for high throughput transcriptome sequencing is the construction of a cDNA library representative of all RNA species.

Several protocols have been developed so far, but their applicability remains tightly linked to the specific platform used. These protocols for cDNA library construction generally involve the following steps: rRNA removal or poly(A) RNA selection, RNA retrotranscription, synthesis of the second strand and, in some cases, amplification of the double-stranded cDNA species. However, this strategy has the drawback of producing a population of fragments smaller than 2 Kb; as a result, the fragmentation process used in the standard 454 GS FLX (Roche) shotgun procedure (nebulization), may not give optimal results since it is more suitable for high molecular weight molecules.

Alternative protocols account for RNA fragmentation followed by retrotranscription. The nebulization step is therefore omitted, albeit the crucial RNA fragmentation step may easily result in RNA degradation.

One recurrent problem in the cDNA library construction is represented by the starting amount of RNA, which is often exiguous. In such conditions, low-expressed transcripts may be scarcely represented despite their considerable biological significance.

Another pivotal element for cDNA libraries preparation, often overlooked in current protocols, is the preservation of the strand specificity information, needed to distinguish either sense and antisense transcripts, or the correct strand of synthesis of other non-coding RNAs, useful to define more precisely the exact boundaries of adjacent genes transcribed from opposite strands, and to determine the levels of expression of coding and non-coding RNA molecules transcribed from overlapping sequences. Indeed, strand-specificity information is useful to correctly map reads that do not contain polyA tails or splice junctions and represent a challenge in the annotation step of RNA-seq reads.

In this work we describe a new unique protocol (Patent pending RM2010A000293-PCT/IB2011/052369) to generate and amplify a representative and strand-specific cDNA library for RNA-Seq applications that may be implemented with all the major platforms currently available (Roche 454, Illumina, Solid). This method is reproducible, fast, easy-to-perform and allows to start from low input total RNA. Moreover, we also provide a suitable bioinformatics pipeline for the analysis of the sequences thereby produced.

Robustness of the strategy here presented was tested by analyzing the transcriptome of two well characterized xenograft tumor masses (OST), sharing the same nuclear genome, but carrying a different status of mitochondrial heteroplasmy [14] and derived from the injection in nude mice of an osteosarcoma cell line. The transcriptome sequencing of the two samples was performed by using two of the main sequencing platforms available on the market: the 454 Roche platform and MiSeq Illumina platform [14].

## Results

**Figure 1:**
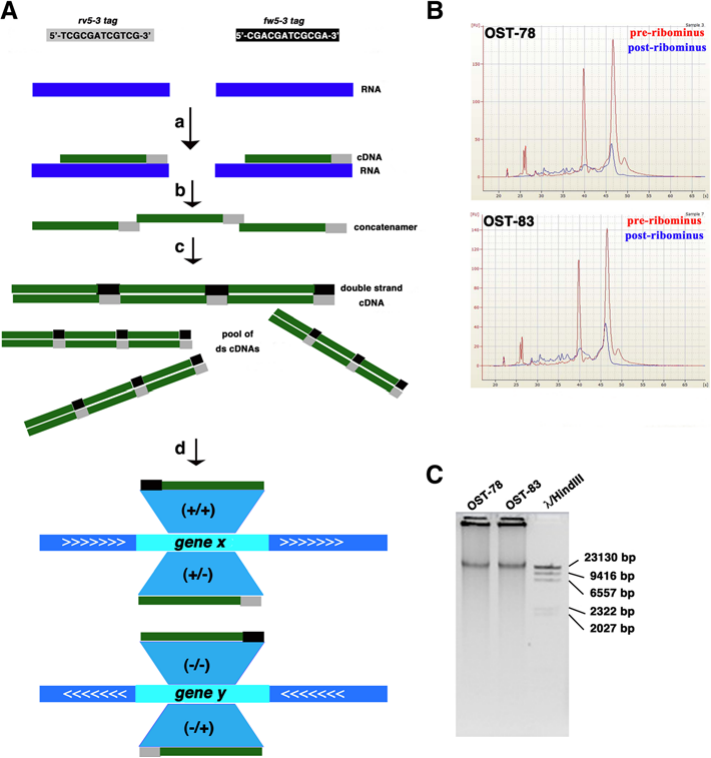
**Schematic representation of the method. A**. Library preparation, starting from deprived ribosomal fraction of total RNA. The two strand-specific tags, *rv5-3* and complementary fw5-3 tag (respectively in grey and black) are represented. a) Synthesis of the 1^st^ cDNA strand, using the Tag-random-octamer primer rv5-3tag. b) 1^st^ strand cDNAs ligation. c) Synthesis of the 2^nd^ cDNA strand with random primers and Phi29 DNA polymerase amplification. This step produced the *fw5-3* tag. d) Mapping of the reads using strand-specific tags to correctly assign reads onto the genome. If the gene x is on the positive strand (+) of the genome, whereas the gene y on the negative strand (-), reads tagged with *fw5-3* tag map gene x on strand + and gene y on strand -. Conversely *rv5-3* tagged reads map an opposite orientation in the same locus. **B**. Removal of ribosomal RNAs from total RNAs. Efficiency of rRNA depletion was evaluated by means of an Agilent 2100 Bioanalyzer using the Agilent RNA 6000 Nano Kit. Both in the OST-78 and in the OST-83 samples, a dramatic reduction in 18S and 28S rRNA bands compared to the pre-Ribominus samples was confirmed. **C**. OST-78 and OST-83 RNA samples were retro-transcribed, ligated and amplified with the Phi29 DNA polymerase. After 4 h amplification, we obtained DNA fragments of high molecular weight (about 23 kb) visualized on 1% agarose gel.

Our approach enables the preparation of cDNA libraries to be sequenced on all the most commonly used massive sequencing platforms currently available on the market. Our method includes the following steps: a) rRNA removal from total RNA and retrotranscription of the rRNA-depleted RNA to cDNA with custom designed 5′ phosphorylated Tag-random-octamers (rv5-3 tag) capable of preserving strand information; b) single-strand cDNAs purification and ligation; c) amplification of the purified cDNAs with the Phi29 DNA polymerase, which is characterized by high strand-displacement, proof-reading activity, high processivity and yields (Figure 1A) [15–17].

To illustrate the validity of our method, we prepared two cDNA libraries starting from two cybrid-derived xenograft tumor masses, which shared the same nuclear genome and were characterized by a different status of mitochondrial heteroplasmy (OST-83 and OST-78 samples, see Methods for details) [14].

500 ng of total RNA of OST-83 and OST-78 samples were initially depleted of ribosomal RNA component. The efficiency of removal of rRNAs from the total starting RNA in both samples was evaluated with the Agilent 2100 bioanalyzer using the specific chip for RNA (Figure 1B). We verified that, starting from 400–500 ng of total RNA, it is possible to obtain around 30–40 ng of RNA depleted of the ribosomal fraction, which is sufficient for the subsequent generation and amplification of a representative and strand-specific cDNA library. Starting from an amount of cleaned RNA <30 ng increased the risk that transcripts with a low number of copies might be only partially represented or absent. This in turn may not ensure high reproducibility in the cDNA amplification (data not shown).

Even poor quality RNA samples with low RNA Integrity Number (RIN) (8 < RIN > 4) could be used for sequencing (data not shown).

5′-phosphorylated Tag-random-octamer custom designed primers (5′-TCGCGATCGTCGNNNNNNNN-3′) were used for the retrotranscription. The trinucleotide TCG, upstream and downstream of the rare-cutter enzyme Pvul restriction site sequence (underlined) ensured that strand information would be preserved. Such trinucleotide was selected because it had the rarest occurrence in the RefSeq *Homo Sapiens* collection, representative of the human transcriptome. Analogously, the 12nt long sequence, composed by the Pvul site flanked at both sides by the trinucleotide 5′-TCG-3′ also resulted to be considerably rare. The need for a tag sequence at the 5′ of the random primer is related not only to the retrieval of the strand information, but also to the protocol-specific step of ligation of single-stranded cDNA molecules into ~ 20 kb long concatenamers (Figure 1A-c). Moreover, the presence of the random nucleotides at the 3′ end of the Tag-random-octamers primers ensured retrotranscription of all RNA species, including those lacking the poly(A) tail, such as replication-dependent histones mRNAs [18].

High molecular weight DNA species for both samples (OST-83 and OST-78) were hence efficiently obtained, quantified with Quant-iT PicoGreen dsDNA Reagent kit (Invitrogen) and visualized by agarose electrophoresis (Figure 1C). An average of 30 μg of ds cDNA were obtained for each library and were suitable for the subsequent fragmentation process required by both 454 Roche protocol or by Illumina Nextera XT protocol.

### 454 and Miseq RNA-seq yield

The number of sequenced reads obtained from the two RNA-seq experiments (Table 1) was very close to the values corresponding to the highest performances of the 454 GS FLX pyrosequencer and of the MiSeq platforms, as indicated by the manufacturer. An average of 500,000 reads and 3,700,000 reads *per* sample was produced respectively within the 454 and the paired-end (PE) MiSeq RNA-seq experiment, with a mean read length of 320 nt (454) and 217 nt (MiSeq) (Table 1 and Additional file 1: Figure S1).

**Table 1:** **Yield of OST samples sequencing and quantification of tagged reads**

sample (sequencing technology)	n.***passed filter*** reads	average read length (nt)	n.of reads with ≥ 1 tag	n. of reads after the tags elimination ≥ 40nt	n.of reads after the tags elimination ≥ 100nt	n.of reads after duplicates elimination ≥ 40nt
**OST-78** (454)	504,879	312	65,514	538,312	446,877	-
**OST-83** (454)	551,068	336	56,494	582,551	505,315	-
**OST-78 [1]** (MiSeq)	1,595,032	207	208,880	1,716,457	1,381,413	421,927
**OST-78 [2]** (MiSeq)	1,595,032	208	177,262	1,786,470	1,377,657	390,845
**OST-83 [1]** (MiSeq)	2,121,777	214	222,766	2,363,988	1,887,111	640,919
**OST-83 [2]** (MiSeq)	2,121,777	214	195,037	2,331,186	1,882,368	605,078

Summary statistics on general throughput of the 454 and MiSeq sequencing of the OST-78 and OST-83 tumor sample show a similar percentage of tagged-reads per sample (11% on average). MiSeq reads were further subjected to the elimination of duplicated sequences. [1] and [2] indicate the forward and the reverse read dataset of the PE sequencing.

### Tags identification and Read strand orientation

*Tag Find* accuracy was tested on a 454 simulated dataset. 2,992 tags out of 3,072 (~98%) were retrieved (Table 2), whereas the remaining 2% of tags remained undetected, as they accounted for more than one sequencing error within the 12 nucleotides of the tag sequence. Furthermore, the 99.3% of tag orientations (*fw* or *rv*) were correctly predicted.

**Table 2:** **Comparison between the number of simulated tagged reads expected and observed**

Sample	N.of reads with 1 Tag	N.of reads with 2 Tags	N.of reads with 3 Tags	N.of reads with 4 Tags	N.of reads with 5 Tag	N. of total tags
**Expected**	5	100	726	166	5	3,072
**Observed**	0	113	782	105	0	2,992

Simulated reads can contain at most 5 tags *per* sequence. The number of Tags found (observed) *per* read was computed from the *tagspositions.txt* file produced by *Tag Find*.

*Tag Find* was hence applied on 454 and MiSeq datasets of reads, choosing a read length cut-off to 40 nt. All the four type of tags were identified, namely the *fw5-3* and *rv5-3*, referred to 5′→3′ directed sequences derived respectively from the 2^nd^ and from the 1^st^ cDNA strand, respectively (Figure 1A-a,c) as well as the *fw3-5* and *rv3-5* tag, 3′→5′ directed. With both the two sequencing platforms Tag Find recognized the same percentage (11%) of reads carrying at least one tag (Table 1 and Additional file 1: Table S1). This percentage will undoubtedly increase with longer reads (up to 700 bp) that will be generated by the latest series of Roche 454 GS Sequencer, the GS FLX Titanium FLX+. The read length filter applied did not significantly reduce the initial yield of reads obtained (Table 1), as the majority of the first cDNA strands produced were at least 100 nucleotides long, thus suggesting that the retro-transcriptase produced long cDNAs. The majority of tagged reads carried only one tag per sequence with a maximum of 6 tags *per* 454 read and 10 tags *per* MiSeq read (Additional file 1: Table S1). Tags identical, or at least with one mismatch with respect to the correct tag sequence, were the most represented in both the read sets (Additional file 1: Figure S2). In general, 5′→3′ oriented tags were the most represented, whereas 0.1% of 454 tagged-reads and 0.6% of tagged-MiSeq reads was composed by chimeric artefacts identified by the presence of at least one 3′→5′ tag (Additional file 1: Figure S2). Boxplot distributions of quality scores (QS) for 454 sequencing showed that the median QS value *per* base was above 20, up to the first 350 nucleotides in each read. Therefore they were trimmed up to an optimal length sufficient to increase the median QS value *per* base to at least 25.

Although read strand orientation can be inferred for both spliced and poly-A reads, a relevant fraction of total mapped reads remains unassigned, thus any protocol improving strand assignment is advantageous. Indeed, it has been already estimated that the fraction of poly-A reads is about 1% of the total mapped in the case of cDNA library preparation enriched for polyadenylated transcripts (i.e. using an oligo-dT tag for the first strand synthesis) [19]. This fraction is much lower in the case of total RNA sequencing with random primers, as for our 454 and Illumina samples. On the other hand we calculated that the percentage of unspliced reads ranges from 29% to 45% in our samples (see data for each expressed gene in Additional file 2) thus leaving a relevant fraction of unoriented reads whose orientation can be inferred in some cases (Table 1) by using our tag-based protocol. Also, about 17% of expressed genes (see below) were single exon genes (see Additional file 2). Finally, our protocol also allowed the identification of antisense transcripts for a large number of genes (Table 3 and Additional file 2f).

**Table 3:** **Spectrum of different RNA species identified within the OST transcriptomes**

Samples (technology)	genes with poly(A) mRNAs	histone genes with non-poly(A) mRNAs	coding mRNAs	non-coding RNAs*	n.of genic loci with at least 1 read mapped in antisense
**OST-78 (454)**	13,365	33	17,498	1,181	1,239
**OST-83 (454)**	13,569	44	18,165	1,178	1,131
**OST-78 (MiSeq)**	13,837	41	20,412	1,503	2,778
**OST-83 (MiSeq)**	14,318	47	21,290	1,629	3,508

*rRNA sequences were excluded from this calculation.

### Mapping results

On average > 95% of 454 and Illumina tag-removed reads could be mapped on the human genome but only 71% and 87% of all 454 and Illumina reads, respectively, passed our strict quality filters (see Methods). Coding and non-coding genic loci were identified by at least one read covering at least 70nt of their genic locus. A mild increase in total number of genes detected was observed with MiSeq data: 13,389 OST-78 genes and 13,609 OST-83 genes identified with pyrosequencing versus 13,931 OST-78 genes and 14,365 OST-83 genes recognized with Illumina technology (Table 3 and Additional file 2b-c). Overall, this result was perfectly in line with those reported in the literature on the average number of genes expressed in most human and mouse tissues, which varies from 11,000 to 13,000 [11]. Interestingly, the average number of genes detected by 454 and Illumina RNA-Seq data represented 87% and 91% of the full set of expressed genes found by Klevebring et al. in U2 osteosarcoma cell line based on the Ensembl gene annotation (21,146 genes) [20].

Given that our protocol is able to generate reads with strand-specific orientation we identified a number of antisense reads for each gene and their exonic or intronic localization (Additional file 2f). On average, 1.4% of all reads mapping in genic loci in each dataset was found in antisense orientation (see Table 3), mostly of them mapping at level of exonic regions (83%).

\We also calculated the number of all coding and non-coding RNA species detected, by counting the number of RNA-specific NCBI RefSeq accession IDs associated to all RNA isoforms (Table 3). Furthermore, to test the ability of our strategy to identify also non-polyadenylated mRNA species, we downloaded the whole set of 87 human genes encoding for replication-dependent histones (http://www.genenames.org/genefamilies/histones), known to be transcribed in non-poly(A) mRNA species. This set of genes was used to query OST-78 and OST-83 genes in order to retrieve all histone genes covered by at least one read. Intriguingly, with both the two technologies we were able to detect almost the half of the whole set of histone genes with non-poly(A) mRNAs in each OST sample (Table 3 and Additional file 2d-e).

### Differential expression and functional categories enrichment results

**Figure 2:**
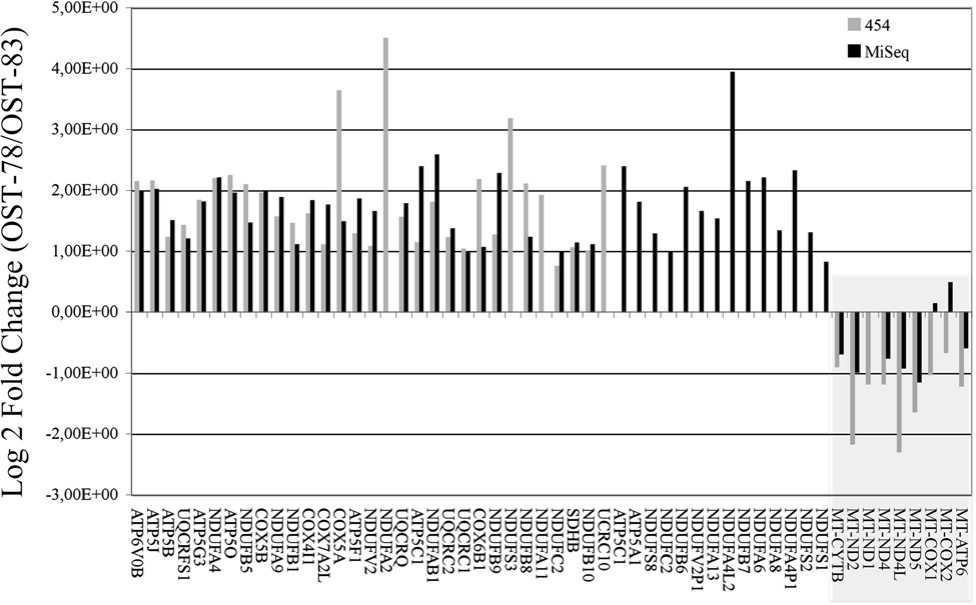
**Differentially expressed RC genes measured by RNA-seq.** Fifty-five log_2_ fold changes of mitochondrial and nuclear differentially expressed genes, encoding for RC subunits. All the genes but two (*MT-COX1* and *MT-COX2*), wherever detected with both the sequencing technologies, showed the identical pattern of up-down-regulation. RC nuclear encoded genes were all over-expressed in OST-78, while the majority of mitochondrial encoded genes (in the grey rectangle) were over-expressed in OST-83.

Fold changes of gene expression were calculated as ratio of digital expression values between the OST-78 and the OST-83 sample (OST-78/OST-83), thus we defined genes with positive log_2_ fold changes as up-regulated in OST-78 and those with negative fold changes as up-regulated in OST-83. Considering that mitochondrial dysfunction was the main feature that distinguished the two samples under study, we specifically looked at the sets of DE genes found with both the NGS techniques (Figure 2) and associated to the mitochondrial Respiratory Chain (RC) assembly (see Methods). Intriguingly, among this group of RC DE genes, all RC DE nuclear encoded genes resulted to be up-regulated in the proliferating and more aggressive OST-78 sample. Conversely the majority of the RC DE mitochondrial encoded genes were significantly over-expressed in the quiescent OST-83 sample (Figure 2 and Additional file 2a).

**Figure 3:**
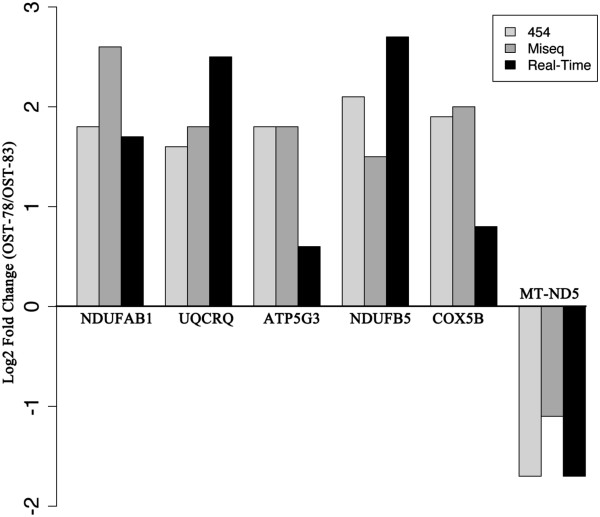
**Comparison of the qRT-PCR and RNA-Seq data.** Log_2_ fold changes calculated with both quantitative Real Time (qRT-PCR) (black) and RNA-seq (light grey for 454 and dark grey for Miseq) analysis are reported. Positive log_2_ fold changes indicate over-expression in OST-78, while negative values over-expression in OST-83. Five differentially expressed RC nuclear-encoded genes, representing four of the five respiratory complexes (I, III, V, IV) were chosen for the validation in qRT-PCR, together with *MT-ND5*, one of the mitochondrial-encoded genes found differentially expressed with both the two ;RNA-seq technologies.

A group of five differentially expressed genes were chosen for the qRT-PCR validation, which confirmed the expression pattern observed in NGS data (RC nuclear genes significantly over-expressed in OST-78 and mitochondrial genes significantly over-expressed in OST-83) (Figure 3). Moreover, a strong Pearson’s correlation between 454 and MiSeq log2 fold changes of expression (calculated as described in Methods) was observed (r = 0.95; CI, Confidence Interval, ranging from 0.55 to 0.99; p-value = 0.004943). Similarly, a high positive Spearman’s correlation coefficient (r = 0.94; p-value = 0.01667) was observed by comparing RPKM values of expression associated to the five differentially expressed genes (Additional file 2a), thus demonstrating that replicable results can be obtained with our method with different NGS platforms. On the other hand, log2 fold change of expression obtained by Real Time showed a weaker correlation with those calculated with NGS techniques (qRT-PCR-454 Pearson’s correlation: r = 0.85, CI ranging from 0.10 to 0.98, p-value = 0.03483; qRT-PCR-MiSeq comparison: r = 0.75; CI ranging from -0.06 to 0.98; p-value = 0.06205). This may be explained considering the low number of genes used for the qRT-PCR assay and also the different way of calculating log2 fold changes adopted for the qRT-PCR and NGS experiments (Methods), that refer to a relative and to an absolute measurement, respectively.

## Discussion

The key aims of the RNA-seq is to catalogue all the RNA species present in a cell, including mRNA and non-coding RNAs, to identify splicing isoforms and to quantify the changing expression level of each transcript under different pathophysiological conditions. Ideally, a method for cDNA library construction should be easy to carry out, quick, high-yield and suitable to obtain a library representative of all RNA species.

All cDNA library preparation protocols currently available provide the utilization of the entire library for a single sequencing run on a single dedicated platform. We here present the development of a unique method that allows the generation of a representative and high-yield cDNA library that can be used for multiple RNA-Seq application with all NGS platforms available on the market, starting from exiguous amounts of total RNA (450-500 ng), not always provided by all protocols. Indeed, the standard Roche cDNA library preparation protocol recommends to start with 200 ng of RNA depleted of rRNA. This implies that it is necessary to have at least 8-10 μg of total RNA, not always available in particular with human samples. Moreover our method allowed to obtain reads in average longer than those obtained with the Roche standard protocol (Additional file 1: Figure S3).

In addition, thanks to the high yields of amplified cDNA library obtained with our protocol (~30-40 μg), it is also possible to reuse the same library also for further subsequent validation experiments as we have here shown. This is of paramount importance because, especially considering the high complexity of the transcriptome disclosed by the ENCODE Project [5], the use of different methodological approaches enabling the qualitative and the quantitative assessment of the transcriptome complexity is presently highly needed. We do also believe that the combined use of Illumina and Roche 454 sequencing platforms may be very effective for transcriptome profiling. In fact, the latter yields longer reads and may therefore provide critical information to assess expressed full-length alternative transcripts, whose quantification may be instead better obtained by using Illumina data. In this context, only a universal protocol such as the one we propose may allow to easily carry out a dual sequencing strategy with a single library efficiently generated even from compromised or partially degraded samples.

An added value is that the Tag-random-octamer primer used in the retro-transcription step, which allows to retain the strand information, can help to optimize the annotation of unspliced reads, and to detect all RNA species, including non-polyadenylated RNAs, which appear to constitute a significant fraction (>25%) of long transcripts present in human cells [21, 22]. Even more importantly, the Tag-random-octamer primers can also be used for metatranscriptomic approaches in order to identify bacterial or viral RNA species in human cells in some physiological or pathological conditions, such as, for example, in the case of multiple sclerosis [23].

Furthermore, the faithful amplification of the library is ensured by singular properties of the Phi29 polymerase: i) strand displacement activity, which enables effective and uniform amplification also in the presence of secondary structures and sequences hardly amplifiable; ii) proof-reading activity among the highest currently available in the realm of high-fidelity Taq Polymerases; iii) high processivity without disassociation from the template, which allows the generation of very long synthesized products, ideal for the nebulization step; iv) high yields of amplified product starting from small amounts of template [15–17]. In addition, the very low percentage of chimeric artefacts observed within the *in silico* analysis and maybe due to the multidisplacement Phi29 amplification, confirms the high proof-reading activity of this enzyme.

The bioinformatics analysis with *Tag Find* allows the user to quantify tagged reads and record all information about their cDNA strand of origin and tags position across the concatenamer. Moreover, the information provided by tags may be used to quantify reads mapped in a discordant orientation with the coding strand, thus belonging to an antisense RNA molecule.

The efficiency of our strategy was tested by using two of the main NGS platforms available: the 454 Roche platform and MiSeq Illumina platform. We sequenced the transcriptome of the two xenograft tumor masses, previously well characterized [14] and derived from the injection in nude mice of an osteosarcoma cell line with a nearly-homoplasmic mitochondrial Complex I disruptive mutation (m.3571insC) in the *MT-ND1* gene. We were able to obtain two representative cDNA libraries of the two tumor transcriptomes, as we identified both poly(A) and non-poly(A) mRNAs, as well as non coding RNAs and even a smaller fraction of antisense RNAs, demonstrating that our method is highly effective in detecting the complexity of gene expression. It is noteworthy that using the cDNA libraries prepared with our protocol, we obtained excellent sequencing results with both the two sequencing platforms. Indeed, the number of detected genes was consistent with the ones estimated in most human and mouse tissues [11] and in the specific U2 human osteosarcoma cell line [20]. To further substantiate these findings, we tried to confirm our results by exploiting the biological significance of the transcriptional profile observed in xenograft samples, therefore we searched only for differentially expressed genes encoding for Respiratory Chain subunits. We found that the expression profile of RC genes well correlated with and may explain the bioenergetic features that we previously investigated in osteosarcoma cells with different loads of m.3571insC *MT-ND1* mutation [14, 24, 25], that influence mitochondrial Complex I assembly. It was therefore not surprising to observe such a distinct pattern of over-expression between nuclear and mitochondrial encoded genes, considering also that the lack of an accompanying up-regulation of nuclear-encoded respiratory genes in the Complex I-depleted OST-83 sample may account for a ‘black-out’ in the nucleus-mitochondrion crosstalk.

## Conclusion

The platform-independent method we proposed for the generation of a representative cDNA library in RNA-seq experiments, combined with a feasible downstream bioinformatics pipeline, provides a powerful tool for whole transcriptome interrogation using single or multiple NGS platforms from which an accurate quantitative and qualitative portrait of complex transcriptome can be traced.

## Methods

### Sample preparation

Two xenograft tumor masses (OST-83 and OST-78), previously characterized in Gasparre et al., 2011 [14], were obtained from the injection of an human osteosarcoma cell line (OSC-83) with a nearly-homoplasmic mitochondrial Complex I disruptive mutation (m.3571insC) in the *MT-ND1* gene, in nude mice. The OST-83 and OST-78 samples carried respectively 83% and 78% of heteroplasmy of the *MT-ND1* frameshift mutation. OST-83 tumor, deprived of Complex I (CI), showed a low-proliferative phenotype, whereas OST-78 tumors, carrying a mutation load below the heteroplasmy threshold for the previously described *oncojanus* anti-tumorigenic effect [14], showed a proliferative, aggressive phenotype and a partial CI assembly.

### RNA isolation and removal of ribosomal RNA (rRNA)

RNeasy Plus Mini kit (Qiagen) was used to extract RNA from snap-frozen tissues. The removal of the ribosomal component from the total RNA was performed using the *RiboMinus Eukaryote Kit for RNA Seq* (Invitrogen), according to the manufacturer’s instructions.

### Primer design and cDNA first strand synthesis

The RNAs, depleted of the ribosomal component, were converted into cDNAs using the “SuperScript III (SSIII) First-strand synthesis system for RT-PCR” (Invitrogen). The associated protocol was modified in relation to some components, times and reaction conditions. In summary, 30-40 ng of post-Ribominus RNA was incubated at 75°C for 5 min with 0.5 mM dNTP and 50 μM 5′-phosphorylated Tag-random-octamers (rv5-3 tag) custom designed (5′-TCGCGATCGTCGNNNNNNNN-3′) in a volume of 10 μl. Samples were placed immediately on ice for 5 min and 1X RT Buffer SSIII, 5 mM MgCl_2_, 10 mM DTT, 40U RNaseOUT, 2.5%DMSO and 200U of SuperScript III Reverse Transcriptase were added. The mixtures were incubated at 25°C for 10 min, followed by 45°C for 90 min and 95°C for 5 min. After blocking the reaction on ice, 1 μl of RNase H was added and incubation performed at 37°C for 20 min.

Synthesized cDNAs were purified using the *MinElute PCR Purification* kit (QIAGEN) to eliminate primers, nucleotides, enzymes and salts. The cDNA mixtures were eluted in 10 μl of EB Buffer (10 mM Tris∙Cl, pH 8.5).

### Second strand synthesis, cDNA ligation and library amplification

The purified cDNAs, resuspended in EB Buffer, were ligated together in long concatenamers, double-stranded converted and subsequently amplified following a modified protocol of the *QuantiTect Whole Transcriptome* kit (QIAGEN).

Since the Invitrogen kit was used for the synthesis of the first strand, the first step of the kit Qiagen was skipped. 10 μl of purified cDNA were mixed and incubated at 22°C for 2 h with 6 μl of Ligation Buffer, 2 μl of Ligation Reagent, 1 μl of Ligation Enzyme 1 and 1 μl of Ligation Enzyme 2. Hence, 29 μl of Amplification buffer and 1 μl of Phi29 DNA polymerase were added and the mixtures were incubated at 30°C for 4 h and then at 95°C for 5 min.

The ligated cDNAs are amplified by REPLI-g DNA polymerase, which moves along the cDNA template strand displacing the complementary strand. The displaced strand becomes a template for replication, allowing high yields of high-molecular-weight cDNA to be generated. This enzyme works as many engineered systems on linearized template [17].

### Library preparation for 454 GS FLX sequencing platform

Sequencing libraries were prepared following the Rapid Library Preparation Method Manual (Roche, GS FLX Titanium series) starting from 1 μg of the ligated ds cDNAs. The libraries thus obtained were amplified in the emulsion PCR system. The enriched-DNA beads of the two samples (OST-83 and OST-78) were deposited onto the wells of a full Roche 454 FLX Titanium Picotiter Plate device and pyrosequenced according to the Sequencing Manual.

### Library preparation for Illumina MiSeq sequencing platform

Sequencing libraries were prepared following the *Illumina Nextera DNA Sample Preparation Method Guide* (Part 15027987 Rev. B, October 2012) starting from 50 ng of ligated ds cDNA. The two indexed libraries thus obtained were applied to cluster generation and a paired-end 250 bp sequencing on Illumina MiSeq platform to obtain about 4 M reads/sample.

### Identification and evaluation of tag strand-specificity

An ad hoc python script, named *Tag Find* (freely available at http://sourceforge.net/projects/tagfind/files/?source=navbar), was developed to clean up the reads from long tags (12 bases) used to identify point of ligations between adjacent cDNA molecules. *Tag Find* uses a *fastq* file as input, and was implemented to discard reads shorter than a user-defined value, after tags elimination. *Tag Find* scans nucleotide windows with length ranging from 11 to 13 nucleotides, starting from the 5′ end of each read, allowing at most one variation (deletion, insertion or mismatch) in the tag sequence. It recognizes the tag sequence in both orientations and saves results. In particular, since the direction of sequence synthesis is 5′→3′, all sequence reads with tags inversely directed (3′→5′) were considered chimeric artefacts produced during the multidisplacement amplification activity of the Phi29 DNA polymerase [26, 27] and can be quantified. Such script was tested on a 454 simulated dataset of 1 K fastq reads (available at http://sourceforge.net/projects/tagfind/files/Test) obtained by using the Metasim software [28]. The dataset was built with reads 350nt long on average and containing at least three tags per sequence (see more details in Additional file 3).

### Reads mapping and genes annotation

Tag removed reads were subjected to sequence quality control with the FastQC program (http://www.bioinformatics.babraham.ac.uk/projects/fastqc). Reads longer than or equal to 40 nucleotides were used for downstream analyses exclusively. Since a drop in median quality score (QS) *per* base was observed only within the 3′end of MiSeq reverse-reads in each sample, these datasets were trimmed using custom python scripts. In particular, upon the observation of quality metrics generated by the FastQC program, OST-78 and OST-83 reverse-reads were trimmed starting from the180^th^ and the 209^th^ (OST-83) nucleotide of the sequence, respectively, in order to maintain a median QS value *per* base ≥ 25, overall the reads. MiSeq SAM files of alignment, generated with the GMAP aligner software [29] using the human reference genome (hg18/NCBI_36), were further subjected to the elimination of duplicated sequences, known to be prevalently produced during the pre-amplification sequencing step of the Illumina workflow [30]. The *MarkDuplicates* function of the Picard tools suite (v.1.68) (http://sourceforge.net/projects/picard/files/picard-tools/1.68) was used to this aim. *Fastq* sequences deprived of duplicates were extracted from SAM files with the *SamToFastq* function of Picard tools and re-mapped onto the human genome through the NGS-Trex platform [31] (http://www.ngs-trex.org). A read coverage of at least 70 bases and a minimum identity of 95% were admitted in this step. In addition, each read was assigned to the gene of origin if at least 50 bases were in overlap with the genomic region annotated as ‘Genic’ in NGS-Trex. Gene annotations were performed by the NGS-Trex platform as described in [31]. At least 95% of nucleotide identity was required to link individual read to specific splicing isoforms, tolerating a trim of 15nt at most. Reads with ambiguous gene assignments were discarded. The presence of rRNAs within each library was further verified considering the amount of reads mapping onto nuclear and mitochondrial human rRNAs identified by NGS-Trex. All coding/non-coding RNA species were identified among the whole set of transcripts mapped, downloaded from the NGS-Trex system, by searching for *NM* and *XM* RefSeq NCBI prefixes for coding and *NR* and *XR* prefixes for non-coding isoforms.

Custom python scripts were used to check on the presence of antisense reads by scanning the *FLAG* field of the SAM file in order to recognize sequences oriented by their tag on the genomic strand opposite to that annotated as ‘Genic’ (see Figure 1A-d for more details).

### Differential gene expression analysis and search of genes coding for the Respiratory Chain subunits

The total reads coverage per gene was used as digital expression value for the differential expression analysis, performed separately on 454 and MiSeq data. All statistical analyses to calculate differentially expressed (DE) genes were conducted within the R environment (http://www.r-project.org). Only genes expressed in both samples were considered and analyzed with the *edgeR* Bioconductor package [32]. In order to eliminate compositional biases of the two cDNA tumor samples compared in the experiment, *CalcNormFactors* function of the edgeR package was used to estimate the normalization factors implemented in the recalculation of each unbiased library size value *per* sample. A stringent adjusted (Benjamini-Hochberg correction) p-value (≤0.01) was chosen to consider a gene differentially expressed. Fold changes of gene expression were calculated with *edgeR* as ratio of digital expression values (OST-78/OST-83). The identification of DE Respiratory Chain (RC) genes was conducted by intersecting the set of DE genes found (data not shown) with the list of RC genes downloaded from the HUGO database (http://www.genenames.org/genefamilies/mitocomplex). RPKM (reads per kilobase per million reads) values [33] of gene expression, calculated with both the sequencing platforms and associated to four nuclear (*NDUFAB1*, *UQCRQ*, *ATP5G3*, *NDUFB5*, *COX5B*) and one mitochondrial-encoded (*MT-ND5*) RC genes, were downloaded from the NGS-Trex platform in order to calculate a Spearman’s rank correlation coefficient and the related p-values of significance.

### qReal-Time PCR

Validation of RNA-Seq data was performed on Phi29 amplified cDNA library (1:50 dilution). Primer sequences were designed on exon-exon boundaries considering the exon regions covered by the reads and using Primer3 software [34]. The presence of 3′ intra/inter primer similarity was ruled out using IDT OligoAnalyzer tool (http://eu.idtdna.com/analyzer/Applications/OligoAnalyzer/) and SNP positions were verified based on the RNA-seq data. The availability of the target sequence was evaluated by prediction of the cDNA secondary structure using Mfold web server [35]. Primer sequences and concentrations used for the Real-time PCR, together with the primer-specific PCR efficiencies are listed in (Additional file 1: Table S3). The PCR reaction was performed with GoTaq® qPCR Master Mix (Promega) and run in 7500 Fast Real-Time PCR System (Applied Biosystems), using the following conditions: 95°C 5 min; 45 cycles of 95°C 15 sec and 60°C 45 sec. The calculations were performed following 2^-ΔΔCT^method. The normalization was performed with geometric average of the 2^-ΔCT^ of two reference genes, namely *U2AF2* and *H2AFV*, which were selected based on RNA-seq data. The normalization of the mtDNA transcription was performed using geometric average of the 2^-ΔCT^ of *U2AF2* and *TUBG1*, the latter being designed on the 3′UTR, in order to normalize for the lack of introns in *MT-ND5.* In order to compare RNA-seq and qReal-Time PCR data, log_2_ fold changes were calculated as ratio OST-78/OST-83. The R coefficients and related CI with a significance level of 0.05 were calculated between log_2_ fold changes of gene expression by applying the Pearson’s correlation.

## Availability of supporting data

*Tag Find* script and the 454 simulated dataset are available at http://sourceforge.net/projects/tagfind/files

454 and MiSeq *fastq* files used for mapping are available at http://193.204.182.50/files/454_Miseq_data.tar.gz

## Electronic supplementary material

Additional file 1: **This file contains (I) Reads length distribution within the two OST samples; (II) Type of tags found within the two samples; (III) Comparison between 454 read length distributions obtained with the Roche standard cDNA library preparation and our protocol; (V) Tags distribution among all the reads sequenced; (VI) Real Time PCR primers sequences.** (DOC 3 MB)

Additional file 2: **The file contains 6 sheets: (2a) Respiratory Chain genes found differentially expressed and related RPKM values; (2b-2c) Number of genes detected with both 454 and MiSeq, in both the OST samples; (2d-2e) Number of histone genes detected with both 454 and MiSeq, in both the OST samples; (2f) Number of genic loci with at least one read mapped in antisense.** (XLS 6 MB)

Additional file 3: **This file contains (I)** ***Tag Find*** **input and usage; (II)** ***Tag Find*** **output description; (III) 454 simulated dataset composition and** ***Tag Find*** **accuracy.** (DOC 32 KB)
