# Supplementary material for: A platform independent RNA-Seq protocol for the detection of transcriptome complexity
Source: BMC Genomics. 2013 Dec 5;14(1):855. doi: 10.1186/1471-2164-14-855 (PMC4046740; doi:10.1186/1471-2164-14-855)
Supplement: Supplementary file 1 — Additional file 1: This file contains (I) Reads length distribution within the two OST samples; (II) Type of tags found within the two samples; (III) Comparison between 454 read length distributions obtained with the Roche standard cDNA library preparation and our protocol; (V) Tags distribution among all the reads sequenced; (VI) Real Time PCR primers sequences. (DOC 3 MB) [file 12864_2013_5558_MOESM1_ESM.doc]

**Additional file 1**

**Supplemental Figures**

**I. Supplemental Figure 1**

**
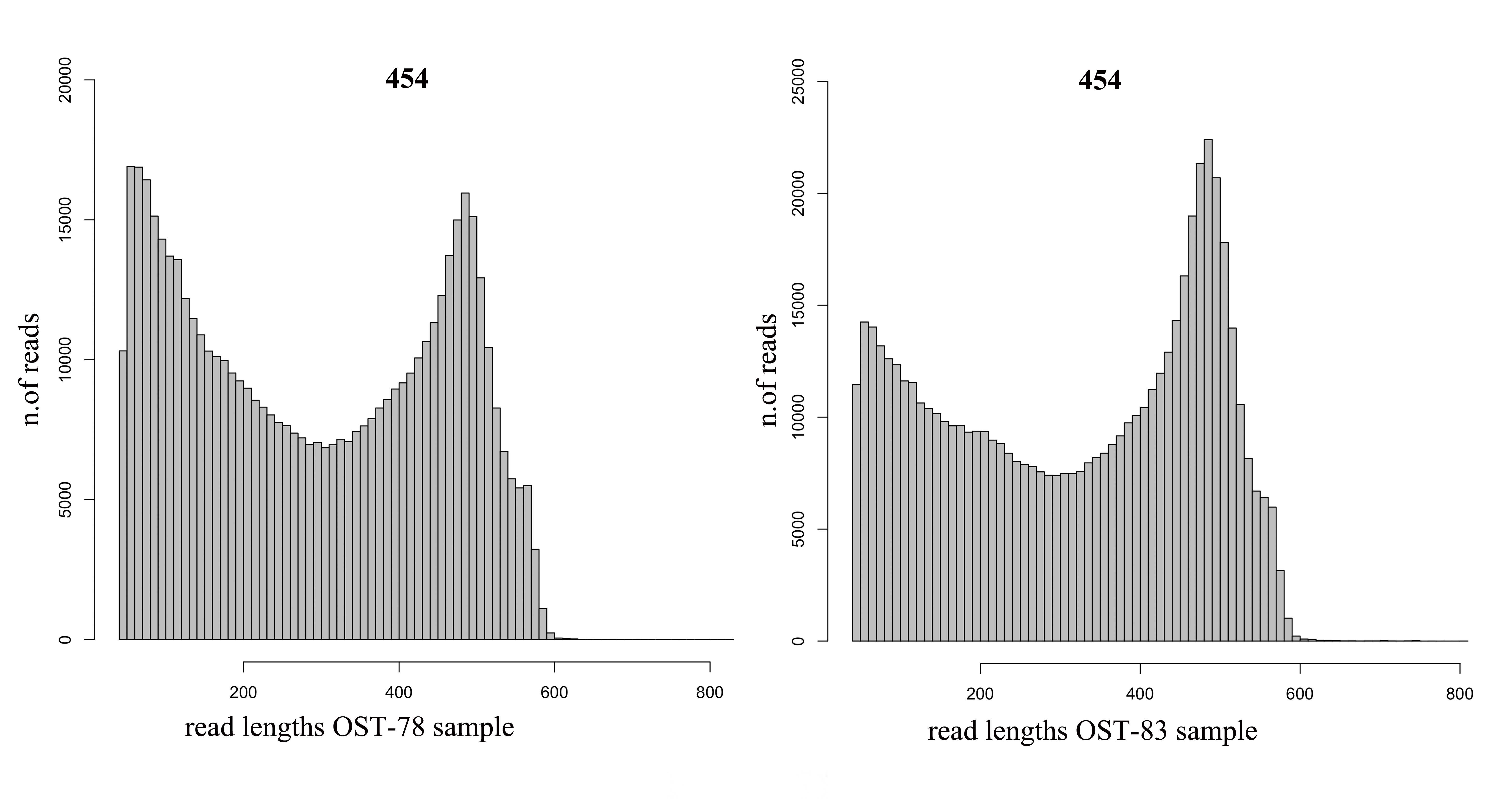
**

**
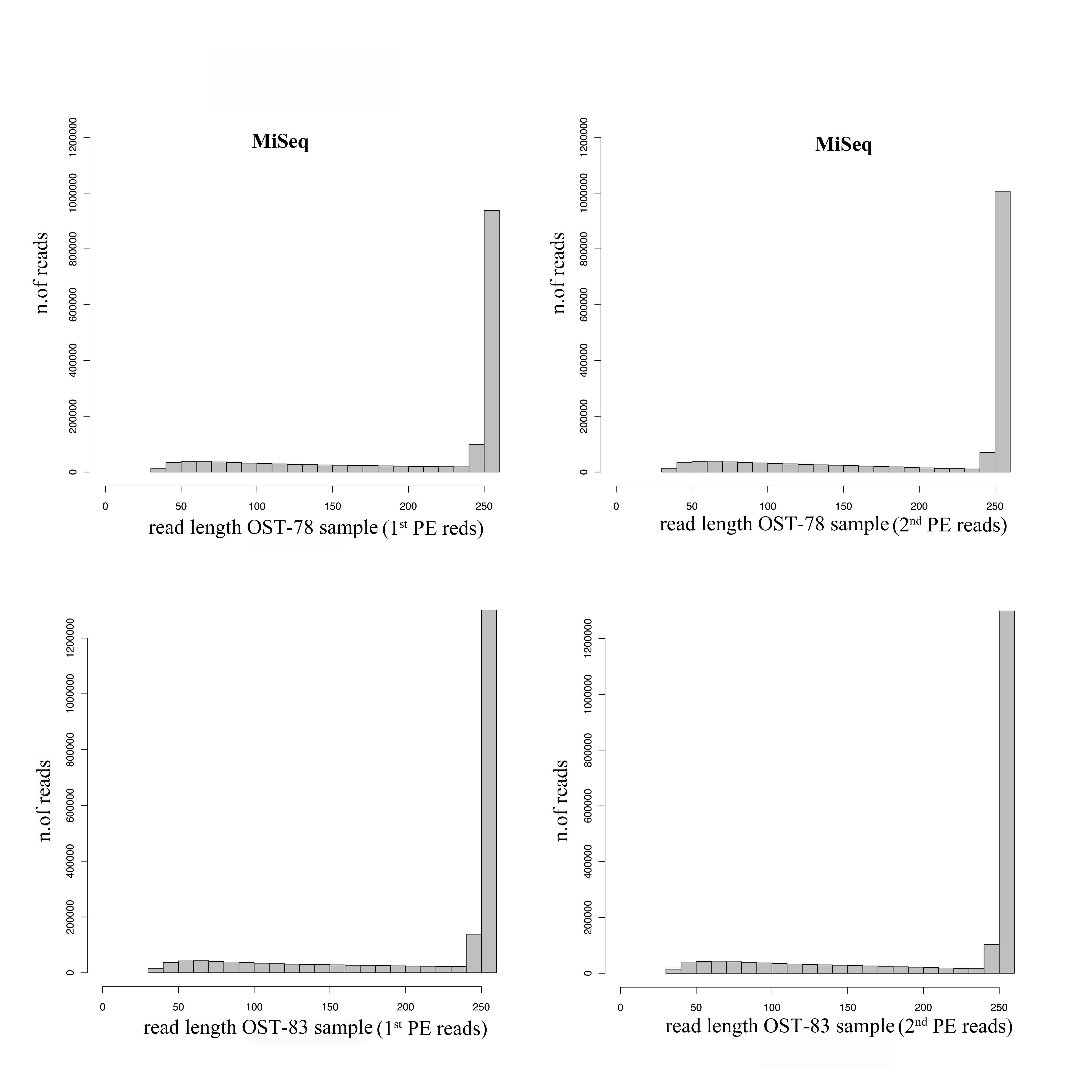
**

**Supplemental Figure 1: Reads length distribution within the two OST samples.**

1^st^ and 2^nd^ refers to the forward and reverse read of the paired-end.

**II. Supplemental Figure 2**

**
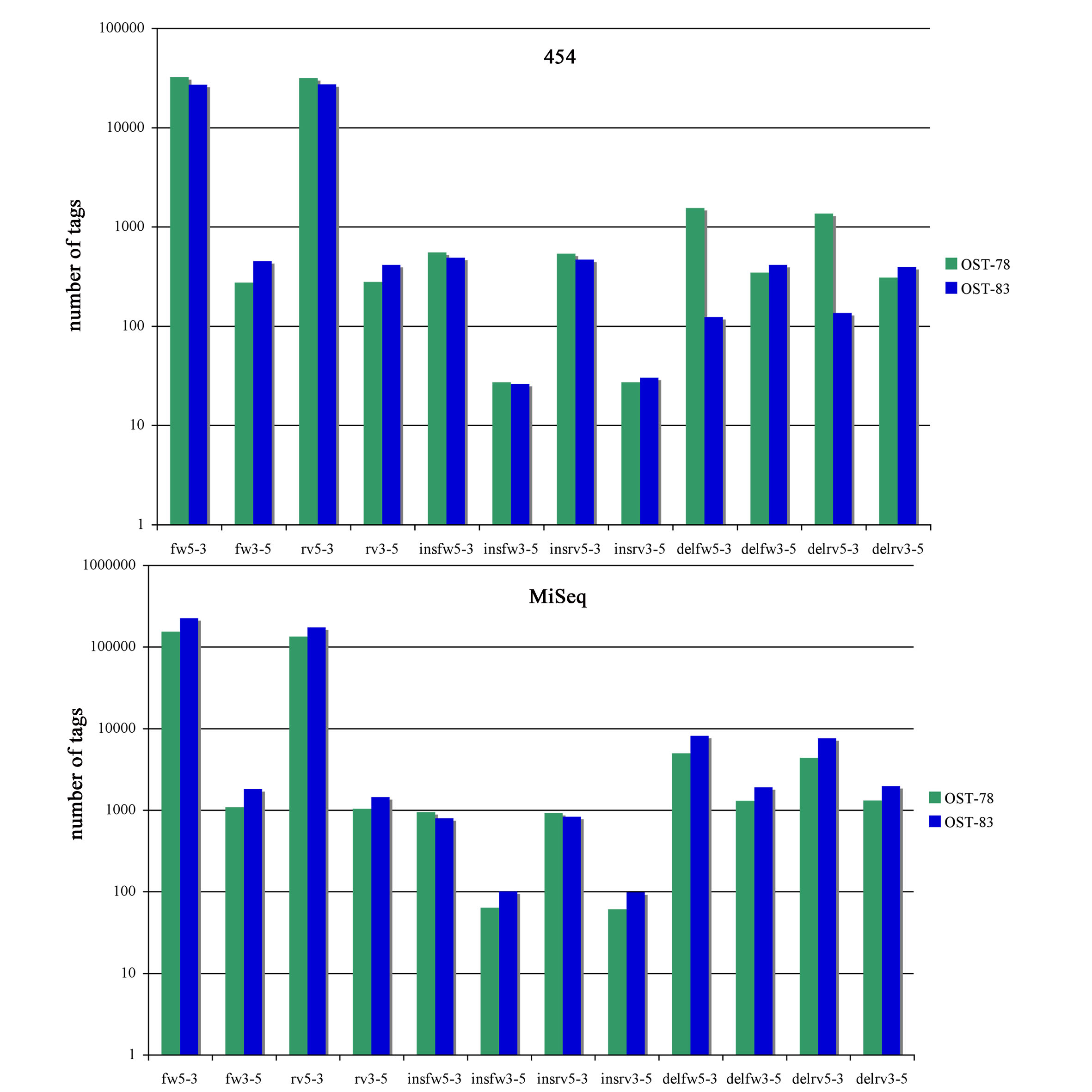
**

**Supplemental Figure 2: Type of tags found within the two samples**. Tags found in forward or in reverse orientation are reported as *fw* ad *rv* respectively, while following numbers indicate the 5’🡪3’ or the 3’🡪5’ DNA strand direction. *Indels* sequencing errors are marked with abbreviations preceding the tag name, i.e. *ins* for insertions and *del* for deletions. A similar distribution of the type of tags found can be observed with both the two sequencing techniques.

**III. Supplemental Figure 3**

**
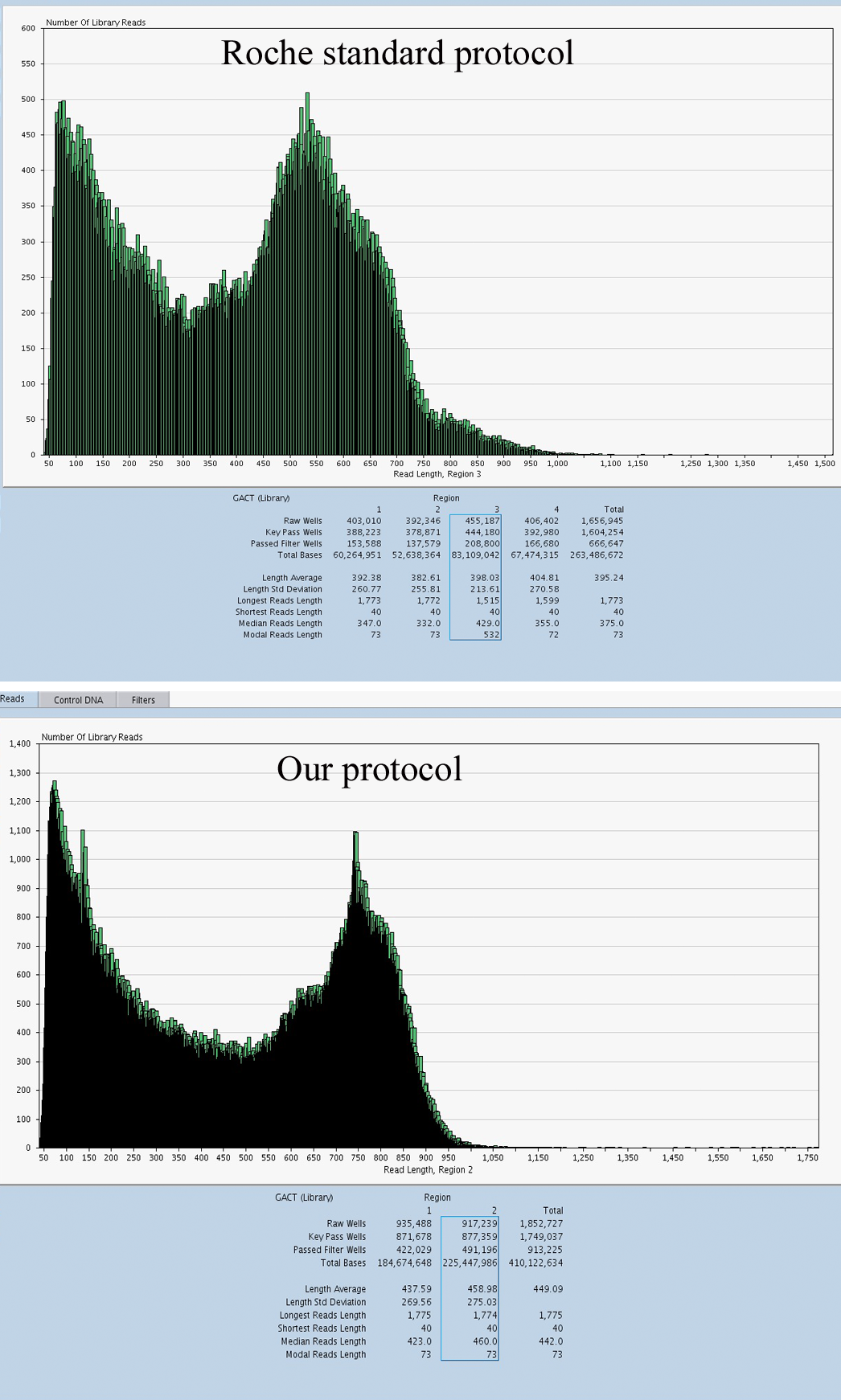
**

**Supplemental Figure 3: Comparison between 454 read length distributions obtained with the Roche standard cDNA library preparation and our protocol.**

cDNA library preparation was carried out on a test sample using the standard Roche protocol and our strategy. Sequencing was carried out with the latest 454 Sequencer series, the GS-FLX+. As reported within each cyan rectangle (region 2 and region 3, respectively), the average read length obtained with our strategy (459 nt) is higher that that obtained with the Roche standard protocol (398 nt).

**Supplemental Tables**

**V. Supplemental Table 1: Tags distribution among all the reads sequenced.**

| **N.of Tags found per read** | **454** | | **Miseq** | |
| --- | --- | --- | --- | --- |
|  | **N.of read**  **(OST-78)** | **N.of read**  **(OST-83)** | **N.of read**  **(OST-78)** | **N.of read**  **(OST-83)** |
| **0 tag** | 439,365 | 494,574 | 2,793,749 | 3,825,751 |
| **1 tag** | 61,911 | 53,351 | 182,131 | 194,110 |
| **2 tags** | 2,784 | 2,145 | 13,522 | 12,758 |
| **3 tags** | 120 | 78 | 1,427 | 1,013 |
| **4 tags** | 93 | 66 | 982 | 914 |
| **5 tags** | 2 | 0 | 37 | 41 |
| **6 tags** | 0 | 1 | 24 | 36 |
| **7 tags** | 0 | 0 | 9 | 11 |
| **8 tags** | 0 | 0 | 20 | 13 |
| **9 tags** | 0 | 0 | 0 | 1 |
| **10 tags** | 0 | 0 | 1 | 0 |

Tags number calculation was based on results reported within the *tagsposition.txt* file created by *Tag Find*. The number referring to each group of MiSeq tags was the result of the sum of tags found within paired-end datasets.

**VI. Supplemental Table 2: Real Time PCR primers sequences**

|  | **Name** | **ENTREZ Gene ID** | **Forward Sequence** | **PCR product length (bp)** | **Conc. in PCR (µM)** | **Slope** | **Eff.** |
| --- | --- | --- | --- | --- | --- | --- | --- |
| RC genes nuclear encoded | ATP5G3_F | 518 | TGCTGCAACAGTAGGAGTGG | 133 | 0.4 | -3.48 | 94% |
|  | ATP5G3_R |  | CAAGGCAAATCCCAGGATAG |  | 0.6 |  |  |
|  | NDUFB5_F | 4711 | CATTGCATTGACTGGGATTC | 102 | 0.4 | -3.525 | 92% |
|  | NDUFB5_R |  | TCTGGGACATAGCCTTCTGG |  | 0.4 |  |  |
|  | NDUFAB1_F | 4706 | CCTGGGCTTAGACAGTTTGG | 103 | 0.4 | -3.442 | 95% |
|  | NDUFAB1_R |  | TGGACACATTAACTTTTCAGCA |  | 0.4 |  |  |
|  | COX5B_F | 1329 | ACTGGGTTGGAGAGGGAGAT | 121 | 0.4 | -3.476 | 94% |
|  | COX5B_R |  | TGGAGATGGAGGGGACTAAA |  | 0.4 |  |  |
|  | UQCRQ_F | 27089 | AGTCTTTCTTTCGCGTGGTG | 152 | 0.4 | -3.405 | 96% |
|  | UQCRQ_R |  | CAGAGACAGGGAACCGTCAT |  | 0.4 |  |  |
| RC gene mitochondrial encoded | MT-ND5_F | 4540 | ATCCTTCTTGCTCATCAGTTG | 157 | 0.4 | -3.403 | 97% |
|  | MT-ND5_R |  | GGCTATTTGTTGTGGGTCTC |  | 0.2 |  |  |
| Reference genes | U2AF2_F | 11338 | CAGTGTTGGCTGTGCAGATT | 132 | 0.6 | -3.254 | 103% |
|  | U2AF2_R |  | GCCTGCGGATCTTTAGTGAC |  | 0.4 |  |  |
|  | H2AFV_F | 94239 | CGTGGTGATGAAGAGTTGGA | 154 | 0.2 | -3.472 | 94% |
|  | H2AFV_R |  | GCTCAGCACACATCCCAGTA |  | 0.4 |  |  |
|  | TUBG1_F | 7283 | CTGTCGCCAGTATGACAAGC | 106 | 0.6 | -3.305 | 101% |
|  | TUBG1_R |  | CTCCCTGGATGTGTCCATCT |  | 0.6 |  |  |

(bp=base pairs; RC=Respiratory Chain; Conc.=Concentration; Eff.=Efficiency)
