# Supplementary material for: A platform independent RNA-Seq protocol for the detection of transcriptome complexity
Source: BMC Genomics. 2013 Dec 5;14(1):855. doi: 10.1186/1471-2164-14-855 (PMC4046740; doi:10.1186/1471-2164-14-855)
Supplement: Supplementary file 3 — Additional file 3: This file contains (I) Tag Find input and usage; (II) Tag Find output description; (III) 454 simulated dataset composition and Tag Find accuracy. (DOC 32 KB) [file 12864_2013_5558_MOESM3_ESM.doc]

**Additional file 3:**

**I. Tag Find input description and usage**

*Tag Find* requires a python interpreter (version 2.5 or higher) and the installation of the Biopython module. *Tag Find* is platform independent tool. The python interpreter and the Biopython module can be downloaded from <http://python.org/> and <http://biopython.org/wiki/Download> , respectively. *Tag Find* uses as input a *fastq* file and an integer value corresponding to the length threshold fixed by the user in order to filter out reads shorter than this value. Tag Find can be launched with this command line:

python Tag_Find.py name_of_the_file.fastq 40

Where 40 is the length threshold used in this example. To eliminate any filtering, a value of 0 must be used.

**II. Tag Find output description**

The *Tag Find* produces two text files: *tagspositions.txt* and *notags.fastq.* The *tagspositions.txt* file reports all the reads IDs, the start position of the tag(s) on the read (if there is no tag, ‘NA’ flag is reported), and a code indicating the type of tag found, i.e. the orientation and direction (*fw5-3/fw3-5* and *rv5-3/rv3-5*) and, if there is an *indel,* a specific label *(delfw/delrv* and *insfw/insrv)* (mismatches do not have a specific label). At the end of each row, read length is reported. The *notags.fastq* file contains all the initial *fastq* sequences cleaned up from tags and selected by the length cut-off defined by the user when launching the script.

**III. 454 simulated dataset composition and Tag Find accuracy**

The Tag Find accuracy in recognizing tags was tested on an artificial dataset of reads obtained simulating a 454 sequencing with the Metasim software. *NM_015074.3*, *NM_006769.3* and *NM_005686.2* human RefSeq mRNAs sequences, all mapping on the plus strand of chromosome 1, were chosen for the artificial library construction. A random selection of 100nt long fragments from the three mRNA sequences was conducted, in order to obtain respectively 35, 45 and 120 fragments, totaling 200 fragments, of equal length. The *fw5-3* tag (5’- CGACGATCGCGA -3’) was added at the 5’ of each fragment and all the 112nt long sequences were linked to form an artificial concatenamer of 22,400 nucleotides with 200 *fw5-3* tags scattered across every 100nt of sequence. The Metasim software was used to simulate a 454 sequencing of the concatenamer. We modified the preset panel of parameters, choosing a uniform model distribution of breaks across the concatenamer for the *in silico* simulation of nebulization. We required 1000 reads as total throughput, a 454-error sequencing model distribution and a mean read length of 350nt (139 cycles). Thus we expected to obtain at least three tags per read. A default quality score value of 40 was associated to each base of the *fasta* output file, in order to produce a suitable input for Tag Find. The simulated dataset is available as (http://sourceforge.net/projects/tagfind/files/Test). We then applied Tag Find to the simulated dataset and we obtained the *tagspositions.txt* file, with all the information about tags position across reads sequences and type of tags found, and the *notags.fastq* file, with all sequences deprived of tags.
